# Gene expression analysis of tumors demonstrates an induction of Th1 type immune response following intratumoral administration of ONCOS-102 in refractory solid tumor patients

**DOI:** 10.1186/2051-1426-2-S3-P230

**Published:** 2014-11-06

**Authors:** Mamun Majumder, Ashwini Kumar, Caroline Heckman, Matti Kankainen, Sari Pesonen, Elke Jäger, Julia Karbach, Timo Joensuu, Kalevi Kairemo, Kaarina Partanen, Tuomo Alanko, Akseli Hemminki, Charlotta Backman, Kasper Dienel, Mikael von Euler, Tiina Hakonen, Juuso Juhila, Tuuli Ranki, Lotta Vassilev, Antti Vuolanto, Magnus Jaderberg

**Affiliations:** 1 Institute for Molecular Medicine Finland, Helsinki, Finland; 2 Oncos Therapeutics, Helsinki, Finland; 3 Krankenhaus Nordwest, Frankfurt/M., Germany; 4 Docrates Cancer Center, Helsinki, Finland; 5 Cancer Gene Therapy Group, University of Helsinki, Helsinki, Finland

## 

Advanced tumors are often immunosuppressive. Intratumoral administration of adenovirus activates Toll-like receptor signalling leading to production of pro-inflammatory cytokines and activation of the innate immune system. Oncolytic adenovirus causes immunogenic cancer cell death and creates a danger signal at tumors, thus undermining immunological tolerance towards tumors. The release of tumor antigens from dying cancer cells results in priming of a potent anti-tumor immune response. This effect may be enhanced by the local production of immunostimulatory molecules coded by the virus. We present results of gene expression analysis of tumors from a Phase I study with ONCOS-102, an oncolytic adenovirus coding for GMCSF, in 12 patients with refractory solid tumors.

A total of 9 intratumoral injections of ONCOS-102 were given to each patient. Biopsies were collected at baseline and 1 and 2 months after treatment initiation. RNA was extracted from fresh-frozen biopsies using standard methods. Gene expression profiling was carried out using the Illumina BeadChip platform. Data was normalized by quantile method, probes presenting the same genes were averaged, and the batch effects were adjusted using ComBat pipeline, as implemented in Chipster (http://chipster.csc.fi/). Finally, log_2 _fold-changes were computed by subtracting baseline data from after treatment data. Network and pathway analyses were conducted through the use of IPA (Ingenuity ^®^Systems, http://www.ingenuity.com). A cutoff value of 2-fold expression change was used to filter differentially expressed genes and run core analysis to identify underlying signaling networks and deregulated molecules.

A significant enrichment of genes involved in immune cell trafficking, inflammatory response and antigen presentation were detected post treatment. A mesothelioma patient showed a prominent post-treatment induction of MAGE3-specific CD8+ T-cells in peripheral blood in IFNγ ELISPOT. Furthermore, a late decrease in metabolic activity was observed in PET imaging 7.5 months after treatment initiation during the follow-up period, measured as a 47% decrease in total lesion glycolysis in comparison to the imaging at 6 months. In the same patient, upstream regulators driving differentially expressed genes detected in the post treatment biopsy included cytokines (INFG, TNF, IL1B, CCL2, CXCL10, IL-17A, CD40LG), enzymes (FN1, NOS2, PTGS2, PARP9), growth factors (BMP2, LEP), kinases (CHUK, CRKL, IKBKB, IKBKG, ITK, STK11, SYK), transcriptional regulators (IRF1, NFATC2, NFKBIA, STAT1, STAT3, ZEB1, RELA), and transmembrane receptors (B2M, CD40, IL6R, TLR2-4, TNFSF1B). Likely, these events collectively induced a higher CD8+ mediated cytotoxic T cell response (GZMB, PRF1) post treatment. Detailed analysis per patient will be presented at the meeting.

## Consent

Written informed consent was obtained from the patient for publication of this abstract and any accompanying images. A copy of the written consent is available for review by the Editor of this journal.

